# Stage-Adapted Cryotherapy-Based Management of Oral Mucosal Melanoma: A Single-Center Retrospective Cohort

**DOI:** 10.3390/jcm15155893

**Published:** 2026-07-28

**Authors:** Yumin Wu, Xinyu Zhu, Zongxi Wu, Yahui Wang, Wanting Jia, Guiqing Liao, Yujie Liang

**Affiliations:** 1 Department of Oral and Maxillofacial Surgery, Guanghua School of Stomatology, Hospital of Stomatology, Sun Yat-sen University, Guangzhou 510055, China; wuym83@mail2.sysu.edu.cn (Y.W.);; 2 Guangdong Provincial Key Laboratory of Stomatology, Guangzhou 510055, China

**Keywords:** oral mucosal melanoma, cryotherapy, immunotherapy, function preservation, survival analysis

## Abstract

**Background/Objectives**: Oral mucosal melanoma (OMM) is a malignancy in which durable local control must be balanced against preservation of speech, swallowing, mastication, and oral structure. Although cryotherapy is a repeatable and function-preserving local modality, its role within contemporary multidisciplinary care has not been clearly defined. This study describes a stage-adapted cryotherapy-based management framework for primary OMM and evaluates survival, local recurrence, and prognostic factors in a contemporary single-center cohort. **Methods**: We retrospectively reviewed 43 patients with primary OMM treated between 2016 and 2026. Treatment allocation was non-randomized and made through multidisciplinary assessment: stage II–III patients received cryotherapy alone, whereas stage IV patients received cryotherapy combined with wide local excision; postoperative adjuvant therapy was considered individually. Overall survival (OS) was estimated using Kaplan–Meier methods, and prognostic factors were explored using univariable Cox regression. **Results:** Median follow-up was 35 months. Estimated 2-year and 5-year OS for the whole cohort were 93.0% and 69.8%, respectively. Local recurrence occurred in 2/11 stage II–III patients and 18/32 stage IV patients. Among stage IV patients, postoperative adjuvant therapy was associated with OS in an unadjusted exploratory comparison (*p* = 0.03); this association may be affected by confounding by indication and selection bias and should not be interpreted as a treatment effect. Nodal metastasis was associated with worse OS in univariable analysis (HR, 3.342; *p* = 0.036). **Conclusions**: This small, single-center, non-randomized cohort describes an institutional stage-adapted cryotherapy-based management framework for OMM. The findings do not establish comparative efficacy or objectively demonstrate functional preservation. Prospective multicenter studies with standardized treatment selection, complete treatment reporting, validated functional outcomes, and quality-of-life measures are required.

## 1. Introduction

Oral mucosal melanoma (OMM) is an uncommon but highly aggressive malignancy, accounting for less than 1% of all melanomas and approximately 0.5% of all oral malignancies [1,2]. OMM often presents at a clinically advanced stage and has historically been associated with poor long-term survival [3,4] because of regional nodal spread, distant dissemination, and delayed recognition [5]. Recognized adverse prognostic factors include nodal metastasis, distant spread, advanced T category, ulceration, tumor thickness, and lymphovascular or perineural invasion [6]. Its location in the oral cavity adds a distinct therapeutic challenge: local treatment must pursue oncological control while preserving speech, swallowing, mastication, dentition, facial contour, and oral competence [7,8]. Thus, the management of OMM is not only a question of tumor eradication, but also a question of how to maintain essential oral function in a disease with limited evidence and heterogeneous clinical presentations.

Wide local excision remains a principal local treatment for resectable OMM [9], particularly for deeply invasive or extensive lesions. However, radical surgery in the oral cavity may require complex reconstruction and can lead to substantial functional and psychosocial morbidity [10]. This clinical reality creates a need for local modalities that can control limited disease while minimizing anatomical sacrifice [11]. Cryotherapy is attractive in this context because it is minimally invasive, repeatable, adaptable to irregular oral surfaces, and can be performed with a margin around clinically visible lesions [12]. These features make it especially relevant for selected superficial, localized, multifocal, or functionally sensitive lesions in which preservation of oral structure is a major treatment goal.

The contemporary treatment landscape has also changed with the increasing availability of immune checkpoint inhibitors and other systemic therapies [13,14,15]. Nevertheless, systemic therapy has not removed the need for effective local control. Responses in mucosal melanoma are generally less predictable than in cutaneous melanoma [16], and persistent or recurrent oral lesions can cause bleeding, pain, infection, dysphagia, and repeated need for salvage treatment [17]. A clinically useful strategy therefore should not frame local therapy and systemic therapy as competing options. Instead, the central question is how a function-preserving local modality such as cryotherapy can be positioned within a multidisciplinary, stage-adapted treatment framework.

Current evidence regarding cryotherapy for OMM remains limited. Most available reports are small, heterogeneous, and do not provide stage-stratified information on survival, recurrence, and systemic treatment integration. This leaves clinicians with insufficient data on which patients may benefit from cryotherapy alone, which patients require combined local treatment, and how local therapy should be coordinated with postoperative adjuvant therapy. Addressing this gap is particularly important in rare tumors such as OMM, where prospective randomized trials are difficult and well-characterized real-world cohorts can provide practical guidance.

The innovation of the present study is the description of a contemporary stage-adapted cryotherapy-based management approach in a relatively large single-center OMM cohort. Rather than treating cryotherapy as a substitute for surgery, this study evaluates its use as part of a structured clinical framework: cryotherapy alone for carefully selected stage II-III disease and cryotherapy combined with wide local excision and individualized systemic therapy for stage IV disease. We aimed to report OS, local recurrence, and exploratory prognostic factors, and to define a clinically actionable role for cryotherapy within multidisciplinary OMM care.

## 2. Materials and Methods

### 2.1. Study Design, Setting, and Patients

**Figure 1:**
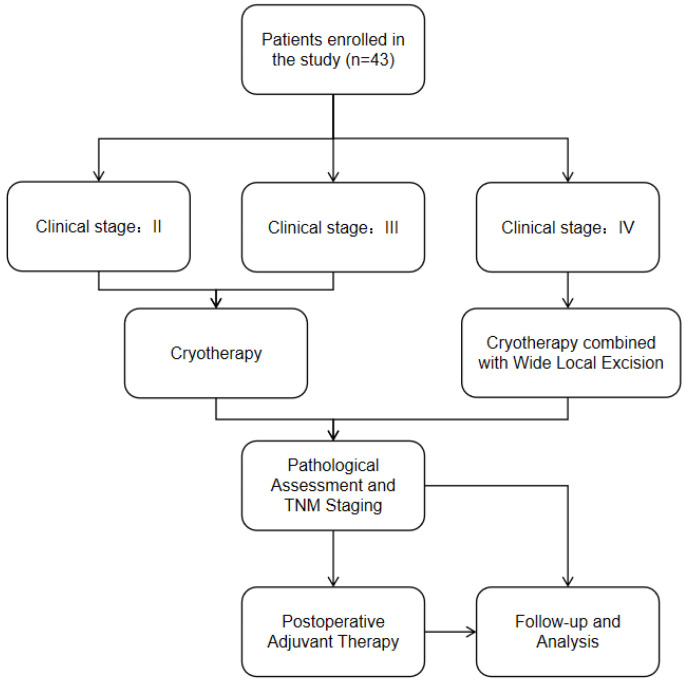
Patient flowchart illustrating study inclusion, stage stratification, and treatment allocation.

This retrospective cohort study included patients with primary OMM treated at the Department of Oral and Maxillofacial Surgery, Guanghua School of Stomatology, Sun Yat-sen University, between January 2016 and January 2026. The study was conducted in accordance with the Declaration of Helsinki and was approved by the Medical Ethics Committee of the Hospital of Stomatology, Sun Yat-sen University (Approval No. ERC-2017-26). Written informed consent for clinical data use was obtained from all patients. Patients were eligible if they had histopathologically confirmed primary OMM and received cryotherapy either alone or as part of combined local treatment. From an initial cohort of 50 patients, 43 were included after excluding seven patients who had no reliable post-treatment outcome information beyond the peri-treatment period (no follow-up data after discharge), making recurrence status and survival status unverifiable. Patients treated with wide local excision alone without cryotherapy were not included in the present analysis. Clinical staging was performed according to the TNM framework of the AJCC 8th edition, as supplemented and refined by the Chinese Anti-Cancer Association (CACA) guidelines for oral mucosal melanoma [18]. While the AJCC 8th edition for head and neck mucosal melanoma only includes T3–T4 categories, the CACA guidelines have incorporated T1 and T2 categories based on extensive clinical data from Chinese patients. This modification allows for more precise stratification of early-stage disease. Demographic, clinicopathological, treatment, and outcome data were extracted from electronic medical records. Prognosis-related parameters systematically collected included lesion morphology [4] (pigmentation, ulceration, size), histopathological features [19] (Breslow thickness, level of invasion, perineural/lymphovascular invasion), and immunohistochemical staining profile [20] (S100, HMB-45, Melan-A). This report follows the STROBE principles for observational studies. The patient inclusion and treatment allocation workflow is illustrated in Figure 1.

### 2.2. Stage-Adapted Treatment Framework

All cases were reviewed by a multidisciplinary team (MDT) including oral and maxillofacial surgeons, medical oncologists, radiologists, and pathologists. The stage-adapted framework was designed to match local treatment intensity to disease extent while prioritizing preservation of oral function when oncologically appropriate. According to the AJCC 8th edition staging and the CACA consensus guidelines, stage II–III patients—whose lesions were superficial (≤5 mm in thickness), well-circumscribed, and anatomically accessible, and for whom wide excision would have incurred significant functional morbidity—received cryotherapy alone. By contrast, stage IV patients—whose primary lesions were deeply invasive (≥5 mm or involving bone), extensive (>4 cm), or rapidly progressive, and for whom surgical resection was anatomically feasible—received a “cryotherapy–wide local excision–cryotherapy” sandwich regimen: pre-resection cryotherapy for debulking and margin demarcation, followed by wide local excision as the primary definitive treatment, and post-resection cryotherapy to address residual superficial disease or satellite pigmentation at margins when further excision would cause functional morbidity. Repeat cryotherapy for metachronous recurrence during follow-up was recorded as salvage treatment rather than initial definitive therapy.

Postoperative adjuvant therapy was considered according to: (1) tumor-related determinants (pathological T category, nodal burden, margin status, lymph vascular/perineural invasion, and metastasis pattern); (2) patient-specific factors (age, ECOG performance status [≤2 generally considered suitable for systemic regimens], organ function, comorbidities, and patient preference after shared decision-making); (3) institutional and logistic availability (drug accessibility, reimbursement policies, and radiotherapy capacity); and (4) alignment with prevailing clinical guidance (AJCC 8th edition, and international melanoma guidelines). Based on this evaluation, the chosen adjuvant regimen could include locoregional radiotherapy [21] (IMRT, 60–66 Gy in 30–33 fractions), systemic immunotherapy [22] (anti-PD-1 agents such as pembrolizumab or nivolumab), cytotoxic chemotherapy [8] (dacarbazine- or temozolomide-based regimens), or targeted therapy [23] (e.g., imatinib for KIT-mutant cases). The timing and sequence were individualized according to wound healing status and clinical response.

Cryotherapy was delivered using a liquid nitrogen dip-stick cryoprobe at approximately −196 °C. The probe tip diameter (3–5 mm) was selected based on lesion size, and the freezing process was guided by real-time visual inspection of ice-ball formation extending 3–5 mm beyond the clinically visible tumor margin. Each session comprised two freeze–thaw cycles: a freezing phase lasting 3–5 min, followed by passive thawing for approximately 2–3 min. The number of sessions per patient was individualized according to lesion size and treatment response, ranging from 1 to 4 sessions (median 2), with 4–6 week intervals between sessions to allow for assessment of response and resolution of post-cryotherapy inflammation. Post-procedural complications, including pain, edema, bleeding, and infection, were managed conservatively with standard analgesics and antibiotics when indicated. Wound healing was assessed at 2 weeks and 4–6 weeks after each session, prior to the next scheduled treatment when applicable. Adverse events were documented and graded according to the Common Terminology Criteria for Adverse Events (CTCAE) v5.0.

### 2.3. Follow-Up and Outcomes

Patients were followed until January 2026 through outpatient visits scheduled every three months during the first five years and every six months thereafter. The primary endpoint was OS, defined as the interval from treatment initiation to death from any cause or last follow-up. Local recurrence was defined as clinically or pathologically confirmed tumor regrowth at the primary site requiring repeat local intervention. Because local control is central to the clinical value of cryotherapy, local recurrence was reported separately for the stage-defined treatment groups.

### 2.4. Statistical Analysis

For descriptive analyses, patients were categorized into three clinical treatment pathways: (1) stage II–III disease treated with cryotherapy alone; (2) stage IV disease treated with cryotherapy combined with wide local excision without postoperative adjuvant therapy; and (3) stage IV disease treated with cryotherapy combined with wide local excision and postoperative adjuvant therapy. Because treatment allocation was non-randomized and strongly related to disease stage and clinical characteristics, these pathways were used for descriptive stratification rather than causal comparison of treatment efficacy.

OS was estimated using the Kaplan–Meier method and compared using the log-rank test. Univariable Cox proportional hazards regression was used to explore associations between clinicopathological variables and OS. Categorical variables were compared using the chi-square test or Fisher’s exact test for small expected frequencies. Multivariable Cox regression and propensity-score methods were not performed because the event count was limited, the number of candidate predictors was large relative to the number of events, and stage was strongly correlated with treatment allocation. These constraints would produce unstable estimates and overfitting. Accordingly, all treatment-related associations are exploratory and should not be interpreted as adjusted causal effects. All tests were two-sided, with *p* < 0.05 considered statistically significant. Analyses were performed using GraphPad Prism 8.0 and IBM SPSS Statistics 27.

## 3. Results

### 3.1. Clinicopathological Characteristics

A total of 43 patients with primary OMM were included. Baseline clinicopathological and treatment characteristics are summarized in Table 1. The cohort included 27 male patients (62.8%) and 16 female patients (37.2%), with a median age of 61 years (range 12–89 years). The most common primary sites were the gingiva (20 patients, 46.5%) and hard palate (13 patients, 30.2%). According to the AJCC 8th edition TNM classification, 5 patients (11.6%) had stage II disease, 6 (13.9%) had stage III disease, and 32 (74.4%) had stage IVA-IVC disease. At presentation, regional lymph node metastasis was present in 17 patients (39.5%) and distant metastasis in 14 (32.6%).

The stage-adapted treatment framework produced two clinically distinct management pathways. Eleven stage II-III patients received cryotherapy alone as a function-preserving local treatment, while 32 stage IV patients received cryotherapy combined with wide local excision as part of multimodality management. During follow-up, local recurrence occurred in 2 of 11 stage II-III patients (18.2%) and in 18 of 32 stage IV patients (56.3%). Recurrent lesions were managed with repeat cryotherapy or local excision when feasible. A total of 89 cryotherapy sessions were performed (median 2, range 1–4). Adverse events were mild to moderate (grade 1–2) in all but one patient: transient pain (88.4%), edema (51.2%), and minor bleeding (34.9%) were most common. One patient (2.3%) developed a grade 3 infection requiring intravenous antibiotics; no grade 4–5 events occurred. Complete wound healing within 4 weeks was achieved in 90.7% of patients; delayed healing occurred only in those receiving multiple sessions for extensive disease. Among 20 local recurrences, 60% were managed with repeat cryotherapy alone, 25% with local excision, and 15% with both; no patient required major reconstructive surgery. Postoperative adjuvant therapy was administered to 13 patients (30.2%), including 6 (46.2%) who received immunotherapy (anti-PD-1 agents), 3 (23.1%) who received chemotherapy (dacarbazine- or temozolomide-based), 3 (23.1%) who received radiotherapy, and 1 (7.7%) who received targeted therapy (imatinib).

### 3.2. Survival Outcomes

**Figure 2:**
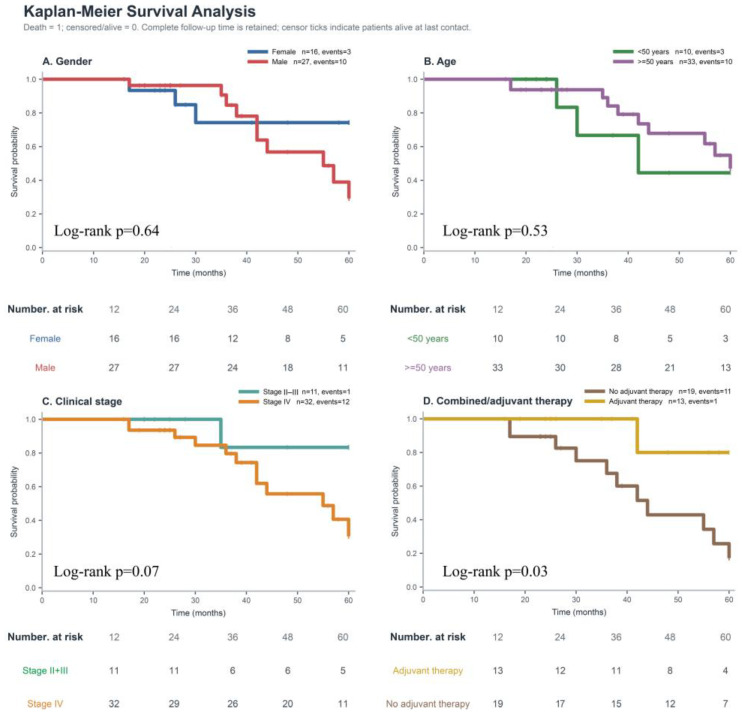
Kaplan–Meier survival curves. (**A**) Overall survival by gender (male vs. female; log-rank *p* = 0.64); (**B**) by age (≥50 vs. <50 years; log-rank *p* = 0.53); (**C**) by clinical stage (stage II–III vs. stage IV; log-rank *p* = 0.07); (**D**) by postoperative adjuvant therapy (Yes vs. No) among stage IV patients (log-rank *p* = 0.03).

The median follow-up was 35 months. For the whole cohort, the estimated 2-year and 5-year OS rates were 93.0% and 69.8%, respectively. Kaplan–Meier survival analysis evaluated the impact of clinicopathological factors on OS (Figure 2). No significant difference was observed between gender (male vs. female, *p* = 0.64; Figure 2A) or age groups (≥50 vs. <50 years, *p* = 0.53; Figure 2B). Patients with stage II–III disease (cryotherapy alone) trended toward better survival than stage IV patients (cryotherapy + wide local excision), though this was not statistically significant (*p* = 0.07; Figure 2C). The Kaplan–Meier estimated 5-year OS was 90.9% (95% CI, 48.4–63.3%, 4 at risk) in the stage II-III group and 62.5% (95% CI, 21.3–24.8%, 4 at risk) in the stage IV group. Among stage IV patients, postoperative adjuvant therapy was associated with OS in an exploratory unadjusted comparison with local treatment alone (*p* = 0.03; Figure 2D).

### 3.3. Prognostic Factors

In univariable Cox regression (Table 2), nodal metastasis was significantly associated with worse OS (HR 3.342, 95% CI 1.083–10.320, *p* = 0.036). Postoperative adjuvant therapy was associated with OS in an exploratory univariable analysis (HR 0.107, 95% CI 0.014–0.825, *p* = 0.032). Distant metastasis showed a trend toward worse OS but did not reach statistical significance (HR 2.971, 95% CI 0.941–9.375, *p* = 0.063). Gender, age, cT classification, and clinical stage were not significantly associated with OS.

## 4. Discussion

This study highlights a clinically important and underreported approach to OMM: integrating cryotherapy into a stage-adapted, function-preserving management framework. Three observations are particularly relevant. First, carefully selected stage II-III patients treated with cryotherapy alone achieved favorable estimated survival and a low local recurrence rate. Second, stage IV disease required combined local and systemic treatment, reflecting the biological aggressiveness of advanced OMM. Third, nodal metastasis and postoperative adjuvant therapy emerged as clinically meaningful prognostic factors. These survival associations should be interpreted with caution given the small early-stage subgroup (*n* = 11), heterogeneous treatment in stage IV patients, and potential selection bias. They reflect an exploratory institutional experience rather than a comparative efficacy assessment. Together, these findings support the concept that cryotherapy can be more than a palliative maneuver; in selected patients, it can serve as a planned local modality within multidisciplinary care.

The most distinctive contribution of this cohort is the early-stage treatment experience. OMM is often managed surgically because of its aggressive behavior [9,24], yet radical excision in the oral cavity can impose major functional costs. The 90.9% estimated 5-year OS and 18.2% local recurrence rate observed among stage II-III patients suggest that cryotherapy may provide a practical balance between oncological control and oral function preservation in selected cases. This observation does not imply that surgery is unnecessary for all early-stage diseases. Rather, it supports a more nuanced management model in which lesion extent, anatomical site, expected functional morbidity, and patient priorities are considered alongside clinical stage.

The functional rationale for cryotherapy is especially important in the oral cavity. Local treatment for OMM may involve the palate, gingiva, alveolar ridge, buccal mucosa, lip, or tongue, and even anatomically successful resection can compromise articulation, swallowing efficiency, mastication, oral continence, dental rehabilitation, and facial appearance [25,26]. These consequences are not minor supportive-care issues; they directly affect nutrition, communication, social participation, and willingness to accept repeated treatment. Cryotherapy offers a tissue-sparing method that can be repeated and can preserve anatomical contours in situations where a wider excision would require reconstruction or produce functional morbidity. The present cohort did not include formal quality-of-life instruments, but the low recurrence rate in the stage II-III cryotherapy-alone subgroup supports the clinical plausibility of using cryotherapy as a function-preserving local treatment in carefully selected patients; functional preservation was not directly measured.

A second functional advantage is the repeatability of cryotherapy. OMM can recur locally or develop multifocal mucosal lesions, and a local modality that can be repeated without exhausting reconstructive options is clinically valuable. In this series, recurrent lesions were managed with repeat cryotherapy or local excision when feasible, illustrating a salvage pathway that may be less disruptive than immediate radical surgery for every local event. This feature is particularly relevant for older patients, patients with comorbidities, and patients in whom oral function is already compromised. Future studies should quantify this benefit using objective and patient-reported endpoints, including speech intelligibility, swallowing function, diet consistency, oral pain, prosthetic rehabilitation, and validated head-and-neck quality-of-life scales.

For advanced OMM, the findings reinforce the need for multimodal treatment. Stage IV patients had higher local recurrence and lower estimated OS than earlier-stage patients, which is consistent with the expected clinical behavior of advanced mucosal melanoma. The association between postoperative adjuvant therapy and improved OS in this subgroup is clinically relevant in the current immunotherapy era [27,28]. Because mucosal melanoma responds less consistently to immune checkpoint inhibition than cutaneous melanoma [29], durable management likely depends on both local disease control and systemic therapy rather than either modality alone. In this framework, cryotherapy may contribute to control of visible oral disease, wide local excision may address extensive local tumor burden, and systemic therapy may target regional or distant microscopic disease. Beyond systemic therapy, radiotherapy remains an important option in many contemporary OMM treatment pathways [21,30], particularly when there is concern for close or positive margins, extensive local invasion, nodal disease, perineural or lymphovascular risk, unresectable locoregional disease, or palliation of symptomatic recurrence. Because radiotherapy was selected through multidisciplinary decision-making and was not uniformly administered, this retrospective cohort cannot determine its independent efficacy. Nonetheless, its role as a locoregional adjunct should not be overlooked, especially in settings where access to immunotherapy may be constrained.

The relationship between cryotherapy and immunotherapy deserves deeper consideration. Immune checkpoint inhibitors do not create tumor antigens; rather, they restore or amplify antitumor T-cell activity that depends on antigen presentation and a permissive immune microenvironment [31,32]. Local ablative therapies, including cryoablation, can induce tumor cell death with the release of tumor-associated antigens, damage-associated molecular patterns, and inflammatory mediators [33,34]. In principle, these events may increase antigen availability to dendritic cells and support T-cell priming [33]. This provides a biologically plausible rationale for combining local cryotherapy with systemic checkpoint blockades, particularly in tumors such as mucosal melanoma where baseline immunogenicity and response to anti-PD-1 therapy are often less favorable than in cutaneous melanoma. However, as discussed below, this immunological rationale remains speculative and requires translational verification.

At the same time, cryotherapy should not be assumed to generate a clinically meaningful systemic immune response in every patient. The immunological effect of freezing depends on the extent and pattern of cell death, local vascular injury, antigen drainage, dendritic-cell activation, and the balance between effector and suppressive immune populations. Incomplete ablation may leave a viable tumor, whereas excessive tissue destruction may create inflammation without effective T-cell priming. The oral mucosa also has a distinctive microbial, vascular, and immune environment, which may influence wound healing and local immune activation [35]. Unlike sharp excision, which physically removes the tumor along with its antigenic material, cryotherapy may leave a zone of treated tissue in situ where tumor antigens and inflammatory signals could, in theory, modulate the local immune microenvironment. However, whether this theoretical difference translates into a meaningful distinction in immune activation between the two modalities remains unknown, as no translational data from this cohort directly address this question. Therefore, the strongest clinical interpretation of our data is not that cryotherapy itself caused systemic immune enhancement, but that cryotherapy can be logically integrated with immunotherapy in a treatment model that addresses both visible local disease and systemic risk.

This distinction is important for advanced disease. In our stage IV subgroup, postoperative adjuvant therapy was associated with OS in an exploratory analysis, while local recurrence remained frequent. These observations suggest that neither local therapy nor systemic therapy should be viewed in isolation. Cryotherapy and wide local excision may reduce the symptomatic and biological burden of oral lesions, while systemic therapy addresses microscopic or overt disseminated disease. A clinically meaningful future direction would be to evaluate treatment sequence. For example, prospective studies could compare cryotherapy before immunotherapy, immunotherapy before cryotherapy, and concurrent approaches, while measuring not only survival but also local immune infiltration, circulating T-cell responses, and local recurrence-free survival.

The biology of mucosal melanoma further supports this integrated perspective. Compared with cutaneous melanoma, mucosal melanoma generally has a lower tumor mutational burden, a different driver mutation spectrum, and a tumor microenvironment that may be less responsive to checkpoint blockade [36,37]. These features help explain why systemic immunotherapy alone may be insufficient for many patients. Local interventions that reduce tumor burden, control bleeding or pain, and potentially alter antigen exposure may be particularly relevant in this setting. Cryotherapy is attractive because it can be delivered directly to accessible oral lesions and can be repeated during surveillance or salvage treatment, making it compatible with the chronic, iterative management often required in rare mucosal malignancies.

The favorable survival observed in this cohort should be viewed as a signal of contemporary integrated care. Historical studies have reported substantially lower 5-year survival for OMM [4,38]. Several factors may help explain the difference, including multidisciplinary decision-making, careful selection of patients for function-preserving local treatment, repeatability of cryotherapy for salvageable local recurrence, and increased availability of immune checkpoint inhibitors. These features are not limitations of the clinical approach; they are part of the evolving real-world context in which rare cancers are now treated. The key message is therefore not that cryotherapy alone changes the natural history of OMM, but that a structured, stage-adapted strategy may help align local control, systemic therapy, and functional preservation.

Nodal metastasis remained an important adverse prognostic factor, consistent with prior mucosal melanoma studies [39]. This finding has practical implications. Patients with clinically node-positive disease require careful baseline imaging, appropriate neck management, and intensive follow-up. The result also supports using nodal status as a key stratification factor in future OMM studies evaluating local and systemic treatment combinations.

The present findings also have implications for how outcomes should be defined in future OMM studies. Survival remains essential, but survival alone does not capture the full value of a local therapy in the oral cavity. A patient who avoids large tissue loss, maintains oral intake, preserves intelligible speech, and remains eligible for subsequent salvage treatment may derive meaningful benefit even when the intervention does not independently determine OS. For this reason, future trials and registries should incorporate local recurrence-free survival, locoregional control, time to salvage treatment, treatment-related pain, wound healing, oral function, and quality-of-life measures. Such endpoints would allow the field to evaluate cryotherapy on the outcomes it is most likely to influence.

This study has limitations inherent to rare-cancer retrospective research. Treatment allocation was stage-dependent and individualized, so the study should be interpreted as an institutional experience rather than a comparative efficacy trial. The sample size and event count limited multivariable modeling. In addition, functional outcomes and quality-of-life measures, which are central to the function-preserving rationale of our treatment framework, were not formally captured, and their absence precludes any direct claim of functional benefit from our data. These issues define the next step for the field: prospective multicenter studies with standardized selection criteria, survival curves with numbers at risk, local recurrence-free survival, translational immune profiling, and validated oral function outcomes. A further limitation is the absence of systematic molecular characterization. The pathology workflow did not routinely test KIT, NRAS, BRAF, NF1, or other driver alterations, and targeted treatment could not therefore be evaluated as a genotype-matched intervention. Recent literature has suggested a distinct molecular profile for oral mucosal melanoma [40,41] that may have implications for targeted therapy selection, but the absence of routine molecular testing in this cohort precludes any inference regarding mutation prevalence or genotype-directed treatment outcomes. Diagnostic confirmation was based on available histopathological and immunohistochemical records; however, PRAME was not routinely performed for all cases, and representative H&E/immunostaining images are not included in the present dataset.

Despite these limitations, the study provides a practical framework for a rare and difficult disease. It positions cryotherapy as a function-preserving local option for selected early-stage OMM and as one component of multimodality care for advanced disease. The deeper clinical value of this framework is that it moves beyond a binary choice between radical surgery and systemic therapy. Instead, it supports individualized treatment planning that considers stage, disease burden, local control, immunotherapy integration, salvageability, and oral function together.

## 5. Conclusions

This small, single-center, non-randomized retrospective cohort describes an institutional stage-adapted cryotherapy-based management framework for OMM. In selected stage II–III patients, cryotherapy alone was associated with descriptive survival and local-recurrence outcomes; these findings do not establish comparative oncologic efficacy or objectively demonstrate functional preservation. In stage IV disease, postoperative adjuvant therapy was associated with OS in an exploratory unadjusted comparison, but this association may be affected by confounding by indication and selection bias. The findings support further prospective evaluation of treatment selection, local control, safety, oral function, and quality of life rather than definitive claims of treatment effectiveness.

## Figures and Tables

**Table 1:** Clinicopathological and treatment characteristics of the study cohort (*n* = 43).

Variable	Number	Percent
Gender Male Female	27 16	62.79% 37.21%
Age in years <50 ≥50	10 33	23.26% 76.74%
Oral lesion		
Gingiva Hard palate Buccal mucosa Lip Tongue	20 13 4 5 1	46.51% 30.23% 9.30% 11.63% 2.33%
TNM classification		
Tumor T2 T3 T4	5 6 32	11.63% 13.95% 74.42%
Node N0 N1 N2	26 16 1	60.47% 37.21% 2.33%
Metastasis M0 M1	29 14	67.44% 32.56%
Clinical stage II III IVA IVB IVC	5 6 14 8 10	11.63% 13.95% 32.56% 18.60% 23.26%
Postoperative adjuvant therapy Yes No	13 30	30.23% 69.77%
Treatment type (among the 13 stage IV patients)		
Immunotherapy (anti-PD-1)	6	46.15%
Chemotherapy	3	23.08%
Radiotherapy	3	23.08%
Targeted therapy	1	7.69%

**Table 2:** Univariate Cox regression analysis of prognostic factors for 5-year overall survival.

Variable		Univariate Analysis	
	5-Year OS (%)	HR (95% CI)	*p* Value
Gender (Male vs. Female)	62.96/81.25	2.035 (0.555–7.457)	0.284
Age (≥50 vs.< 50)	69.70/70.00	0.665 (0.181–2.449)	0.540
cT classification (T4 vs. T2-T3)	62.50/90.90	5.288 (0.680–41.152)	0.112
cN classification (N1-2 vs. N0)	64.71/73.08	3.342 (1.083–10.320)	0.036
cM classification (M1 vs. M0)	57.14/75.86	2.971 (0.941–9.375)	0.063
Clinical stage (Stage IV vs. Stage II-III)	62.50/90.90	5.288 (0.680–41.152)	0.112
Postoperative adjuvant therapy (Yes vs. No)	92.31/60.00	0.107 (0.014–0.825)	0.032

OS overall survival, CI confidence interval, HR hazard ratio.

## Data Availability

The data presented in this study is available on request from the corresponding author.

## Abbreviations

The following abbreviations are used in this manuscript:

AJCC	American Joint Committee on Cancer
CI	confidence interval
HR	hazard ratio
OMM	oral mucosal melanoma
OS	overall survival
PD-1	programmed cell death protein 1
STROBE	Strengthening the Reporting of Observational Studies in Epidemiology
WLE	wide local excision
